# Consensus Statement on Animals’ Relationship with Pediatric Oncohematological Patients, on Behalf of Infectious Diseases and Nurse Working Groups of the Italian Association of Pediatric Hematology-Oncology

**DOI:** 10.3390/jcm12072481

**Published:** 2023-03-24

**Authors:** Giulia Fiumana, Debora Botta, Maria Francesca Dalla Porta, Simone Macchi, Elena Soncini, Antonio Santaniello, Orlando Paciello, Matteo Amicucci, Monica Cellini, Simone Cesaro

**Affiliations:** 1 Department of Medical and Surgical Sciences of the Mothers, Children and Adults, Post Graduate School of Pediatrics, University of Modena and Reggio Emilia, 41121 Modena, Italy; 2 Pediatric Unit Ospedale Santissima Annunziata di Savigliano, 12038 Savigliano, Italy; 3 Pediatric Oncology Unit, Fondazione IRCCS Istituto Nazionale dei Tumori, 20133 Milan, Italy; 4 Pediatric Oncohematology and Bone Marrow Transplant Unit, Children’s Hospital, Spedali Civili, 25123 Brescia, Italy; 5 Department of Veterinary Medicine and Animal Production, Università degli Studi di Napoli Federico II, 80137 Naples, Italy; 6 National Federation of Italian Veterinarians (FNOVI), 26100 Cremona, Italy; 7 Department of Onco Haematology and Cell and Gene Therapy, Bambino Gesù Children’s Hospital, IRCCS, 00165 Rome, Italy; 8 Pediatric Hematology Oncology Unit, Azienda Ospedaliero Universitaria Policlinico di Modena, 41125 Modena, Italy; 9 Pediatric Hematology and Oncology, Department of Mother and Child, Azienda Ospedaliera Universitaria Integrata, 37126 Verona, Italy

**Keywords:** animal-assisted therapy, zoonoses, infectious complications, pediatric oncology, chemotherapy, stem cell transplantation, immunocompromised host, one health, children health, quality of life

## Abstract

Contact with animals in pediatric oncohematologic patients is associated with many benefits, but the risk of contracting zoonoses, even if low, must be considered by clinicians. In order to assess the awareness about this topic, we surveyed the Italian pediatric oncohematology centers, which resulted in heterogeneous responses. The Infectious Diseases Working Group and the Nurse Working Group of the Italian Association of Pediatric Hematology-Oncology, together with veterinarians from the National Federation of Italian Veterinarians, drew up a consensus document to unify the indications to be given to families with the aim of guaranteeing a safe interaction between patients and animals and improving the collaboration of clinicians with veterinarians and families.

## 1. Introduction

The prognosis of childhood cancer has improved in recent decades, with overall survival rates exceeding 80% in high-income countries [1]. This success has led to intensifying efforts to ensure a higher quality of care, both during treatment and for long-term survivors.

At diagnosis, families are informed about the disease, treatment, and possible complications, including precautions to be taken during periods of immunodepression. In this context, parents and patients often ask healthcare professionals for advice about animals that are already part of the family or about adopting a puppy as a playmate for the child facing a period of isolation from peers. In recent years, complementary treatments have been implemented with Animal-Assisted Interventions (AAI) [2,3]. Although the importance of informing families about how to deal with animals is recognized, reference guidelines are lacking. In order to improve knowledge on this topic, the Infectious Diseases Working Group (IDWG) and the Nurse Working Group (NWG) of the Italian Association of Pediatric Hematology-Oncology (AIEOP) proposed a consensus focused on the prevention of zoonoses in immunocompromised oncohematologic pediatric patients.

## 2. Materials and Methods

The members of the Scientific Committees of AIEOP IDWG and NWG planned the consensus in collaboration with experts in zoonoses belonging to the National Federation of Italian Veterinarians (FNOVI).

The project was carried out according to the method of the nominal group technique or expert panel method [4], with the following steps: (a) identification of the members of the Consensus Working Group (CWG) by the Scientific Committees of IDWG and NWG based on their interest in and knowledge of the subject, which identified five pediatricians, three nurses, and two veterinarians; (b) formulation and approval of the consensus topics by the members of the CWG; (c) a search of the literature by the CWG members on the identified topics; (d) proposal and discussion of the consensus statements among the CWG members; (e) approval of the statements by the CWG members; and (f) approval of the consensus statements by the AIEOP IDWG and NWG members. 

The project started in July 2021 and ended in June 2022. The first meeting of the IDWG and NWG to define item “a” was held in person, while the remaining meetings for items “b”, “c”, “d”, “e”, and “f” were held by remote video call. The voting for the approval of the consensus statements among the members of the CWG, IDWG, and NWG was completed by e-mail. 

Considering the different attitudes of the centers towards this issue and the lack of guidelines on this topic, the CWG decided to survey the practices of the AIEOP centers before defining the topics and starting the literature search. The online survey was sent in 2021 and received responses from 22 of the 42 AIEOP centers. Survey questions asked about advice provided to families, use of AAI, incidence, and management of zoonoses. The results of the survey are summarized in Table 1 and showed heterogeneous practices, a lack of common practices, and the desire to receive shared indications to be provided to families.

**Figure 1:**
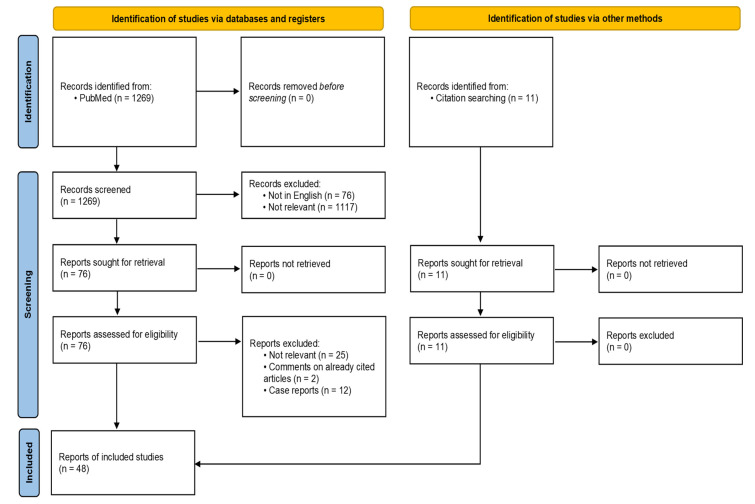
Selection of references using the PRISMA methodology [6].

The literature search on the PubMed database covered the period from 1 January 2000 to 31 December 2021, using the following keywords: ((animal assisted therapy) OR (zoonoses)) AND ((Cancer) OR (Neoplasm) OR (Tumor) OR (Malignancy) OR (bone marrow transplantation) OR (hematopoietic cell transplantation) OR (peripheral blood stem cell transplantation) OR (cord blood transplantation) OR (immunocompromised host)) AND ((domestic animals) OR (animals)). The evaluation of the 1269 references, using the PRISMA methodology [5,6], resulted in the selection of 51 studies (Figure 1).

## 3. Statements and Discussion

### 3.1. What Is the Role of Contact between Children with Oncohematologic Diseases and Animals?

Hospitalization represents a critical factor for cancer patients because it increases stress and causes a reduction in social interactions and changes in daily routine.

It is known that the relationship with animals plays a positive role in improving the quality of life, especially during illness; in fact, it is now generally recognized that the benefits of pet ownership outweigh the risks associated with contact with the animal [7,8]. Activities involving animals are defined as Animal-Assisted Interventions (AAI), including Animal-Assisted Activities (AAA), i.e., informal play and recreational interactions, Animal-Assisted Education (AAE) with educational and promotional purposes, and Animal-Assisted Therapies (AAT) that represent structured therapeutic interventions provided by specialized personnel whose goals and progress are predetermined and individualized [9,10].

The use of AAIs is increasingly being introduced in pediatric oncology, with benefits reported in terms of reduction in pain, stress and anxiety, increased appetite, familiarization with the hospital environment, facilitation of communication, therapeutic alliance, acceptance of hospitalization and invasive procedures, faster recovery from surgery, and overall improvement in quality of life through humanization of care [3,11,12,13,14,15,16,17,18,19,20,21,22,23,24,25,26,27].

*Statement 1:*  *Contact with animals, both domestic and in AAI, has a positive role in improving the quality of life of pediatric patients with oncohematologic diseases and should be encouraged when patients’ clinical conditions allow.*

### 3.2. What Are the Risks Associated with Contact with Animals?

WHO defines “zoonoses” as “those diseases which are naturally transmitted between vertebrate animals and man” [28].

Zoonoses include a wide range of viral, bacterial, fungal, and parasitic infections that can be transmitted by direct exposure (contact with infected animals or their biological fluids), invertebrate vectors (arthropods), environmental exposure (contact with infected feces or urine, inhalation of aerosols) or food contamination (ingestion of contaminated water, milk, or other foods) [2]. It is also important to note that most pathogens responsible for zoonoses are environmental contaminants; therefore, it is possible that the immunocompromised patient and the animal simultaneously acquire the infection through environmental exposure rather than through actual pet-to-human transmission [29].

The most common routes of transmission are the oral route (*Campylobacter* spp., *Cryptosporidium* spp., *Salmonella* spp., *Giardia* spp., *Toxoplasma gondii*) and by inhalation (*Cryptococcus neoformans*, *Chlamydia psittaci*). Particular attention must be paid to bites or scratches for the risk of systemic (*Bartonella henselae, Pasteurella multocida*) or soft tissue (*C. canimorsus* and *C. cynodegmi*) infections; the latter can also be caused by non-traumatic contacts with infected animals (*Mycobacterium marinum*, dermatophytosis) [2].

The incidence of zoonoses in immunocompromised patients is difficult to determine because cases are sporadic and not subject to disease notification [30,31,32,33,34,35,36,37]. Lothstein et al. reported a 0.86% incidence of zoonoses in 10197 pediatric patients with acute leukemia and 1 death from *Cryptosporidium* [38]. Given the benefits associated with the relationship with animals and the significantly low risk of zoonoses, removal from the family is not indicated. However, the immunocompromised state may increase the risk of infection and patients may develop severe zoonoses and more severe complications [8,39,40]. In addition, there is increasing awareness of a group of high-risk resistant bacteria, defined as ESKAPE (*Enterococcus faecium*, *Staphylococcus aureus*, *Klebsiella pneumoniae, Acinetobacter baumanii*, *Pseudomonas aeruginosa,* and *Enterobacter* spp.), that may be transmitted by animals in the AAI setting [41,42,43].

Based on clinical experience, patients with hematologic diseases (leukemia and lymphoma) in the induction phase are considered to be at a higher risk than those with solid tumors and during maintenance therapy. In this regard, a study from Meazza et al. showed that discontinuation of *P. jirovecii* infection prophylaxis with trimethoprim/sulfamethoxazole in patients affected by solid tumors did not increase the risk of infection [44].

Finally, it is important that veterinarians monitor animal health and, when zoonoses are suspected, public health experts should be involved to monitor any epidemic cluster and collect information regarding local epidemiology [45].

*Statement 2:*  *The risk of zoonotic infections is low and depends on the immunosuppression status of patients, species, age of animals, and hygiene. If standard hygiene measures and veterinary indications are followed, the risk of zoonoses is not a reason to prohibit patients’ contact with animals during the treatment phase.*

### 3.3. When and How Is This Topic to Be Discussed with Families?

Healthcare providers should investigate whether pets are present at the time of diagnosis and, if so, provide guidance on zoonoses’ prevention [46]. In this context, the collaboration between healthcare providers and veterinarians is necessary [7,29,43,47,48,49], aimed at keeping animals healthy in a safe environment for themselves and patients, according to the integrated One Health approach [42,50,51,52,53].

Written information should be used, such as booklets containing information on the risks associated with contact with animals, together with advice on precautions, hygiene standards, and any restrictions. More detailed materials should also be prepared for specific needs: families who already own animals, families who are considering bringing an animal into the family, patients at specific phases of the disease, and patients who wish to take part in AAI. The content of the educational material should be shared with experienced veterinarians.

*Statement 3:*  *It is recommended that patients and families be educated on the proper management of companion animals from the time of initial admission. The use of brochures or other materials that have been previously shared with reference veterinarians is recommended to ensure consistency of information within the healthcare group.*

### 3.4. When Families Decide to Adopt an Animal, Are There Any Recommended Species and Others That Are Better to Avoid?

The adoption of a new pet after diagnosis or hematopoietic cell transplantation (in both situations, the first 3-6 months are considered the period at higher risk of infection) is not recommended, but if families decide to acquire a pet, it is recommended to prefer a young/adult animal; in particular, it is recommended to avoid puppies younger than 6 months (less than 1 year in the case of cats), as younger animals may be more susceptible to infections and may not have completed their vaccination schedule. Species with a higher risk of zoonotic transmission should be excluded: dogs or cats younger than 6 months (risk of *Campylobacter* spp. and *Bartonella* spp.); reptiles, amphibians, and exotic species (risk of *Salmonella* spp.); rodents (risk of *Salmonella* spp. and lymphocytic choriomeningitis virus); and young poultry (risk of *Salmonella* spp. and *Campylobacter* spp.) [2,53,54,55,56]. Families with children under 5 years of age should not have pet reptiles (turtles, lizards, snakes), amphibians (frogs, toads, newts, salamanders) or backyard poultry (including chicks and ducklings), and rodents (rats, mice, hamsters, gerbils, guinea pigs) due to the risk of serious disease transmission from harmful pathogens that these animals may harbor [57]. For the same reason, immunocompromised children should avoid contact with stray animals, reptiles, wild birds, and primates; fish can also be a source of skin infections [2,54,55,56].

*Statement 4:*  *The purchase or adoption of puppies, rodents, reptiles, amphibians, and exotic species is not recommended during the first 3-6 months after the diagnosis of oncohematologic disease or hematopoietic stem cell transplantation.*

### 3.5. Are There Specific Indications for Those Asking to Adopt Animals?

Families should be guided by a veterinarian in selecting animals from a controlled origin to avoid common atypical mycobacteria or other infections in puppies imported through illegal trade [58]. All new pets from pet stores, breeders, or shelters must be examined by a veterinarian before being introduced into the domestic environment [2,59]. If the family decides to acquire an exotic animal, it is necessary to consult a veterinarian with expertise in the species to ensure adequate monitoring of the animal’s health [47]. It is important to carry out annual routine visits and the vaccination schedule required for each species, but it must be remembered that dogs and cats are vaccinated against species-specific pathogens and not for protection against zoonoses; none of the vaccines used for dogs and cats are expected to cause problems for children receiving chemotherapy, except for those against *Bordetella* spp. (used to prevent dog cough). Children being treated for cancer should avoid contact with dogs that have been given the *Bordetella* vaccine within the last 6 weeks [60].

*Statement 5:*  *It is recommended to select animals from controlled breeders and to carry out health checks before introducing them into the family.*

### 3.6. Is Here Any Hygiene Advice for Those Who Already Own Animals?

Adult supervision is always recommended during the interaction between animals and children [2]. It is essential to always wash hands after contact with animals, especially if there is contact with the animal’s saliva and/or feces [2,53,54,58,59,61,62]. Contact with animals should be avoided if they have diarrhea or if a disease is suspected [2,54,62,63], and a veterinarian should visit the animal and perform culture tests on its feces to exclude *Cryptosporidium, Salmonella*, or *Campylobacter* infection [2]. In the case of bites or scratches, wounds must be carefully washed under running water and disinfected with chlorhexidine, and the reference oncohematology center should be notified for possible antibiotic prophylaxis [2,53,59]. To reduce this risk, animals’ nails should be kept short, and rough play should be avoided [2].

Animals should be fed high-quality products and drinking water, and home-prepared food should be cooked and/or pasteurized [2,43,53]. Animals should be kept indoors to avoid hunting and contact with uncontrolled or waste food and feces from other animals.

Meticulous daily hygiene is fundamental: coat cleaning is essential to maintain skin integrity and healthy hair; kennels, litter boxes, cages, and toys must be carefully cleaned by immunocompetent people; and feces must be sealed in plastic bags. Litter boxes must be kept away from pet food bowls and should be disinfected at least once a month [2].

*Statement 6:*  *Hand washing is always recommended after contact with animals. The nutrition and hygiene of animals must be constantly monitored; hygiene must be carried out by immunocompetent subjects and not by patients.*

### 3.7. Is Contact with Animals Outside the Home Allowed?

Contact with animals in non-domestic environments may involve interaction with animals of unknown health and hygiene status and, hence, should be avoided [2]. The animals of friends or relatives must meet all the requirements described above to allow visits to immunocompromised patients.

*Statement 7: Contact with non-domestic animals whose health and hygiene conditions are uncertain should be avoided.*

## 4. Conclusions

Zoonoses in immunocompromised pediatric patients are rare but potentially dangerous, so prevention is important. The limited review available in the medical literature on this topic did not allow the publication of evidence-based guidelines, so we produced general recommendations based on current knowledge and expert opinion. Importantly, many studies showed benefits from the patient–animal relationship, so the implementation of AAI practices should be promoted to improve the quality of care for pediatric oncohematology patients. Through our work, we encourage closer collaboration between patients, families, healthcare providers, and veterinarians to ensure a safe and happy relationship with animals.

## Figures and Tables

**Table 1:** Results of the survey submitted to the AIEOP centers.

Indications to Patients	% (*N*)		% (*N*)	
At your center, are families informed about the risks of zoonoses, and are they given indications for prevention?	Yes 95.5% (21)		No 4.5% (1)	
Information provided is derived from:	Experts’ indications 9.5% (2)	Literature 9.5% (2)	Comparison with other centers 4.8% (1)	All of the above 76.2% (16)
How is the information provided?	Brochures 33.3% (7)		Orally 66.7% (14)	
Who is in charge of giving the information?	Medical staff 68.2% (15)	Nurses 18.2% (4)	Both medical staff and nurses 13.6% (3)	Veterinarians 0 (0%)
When is the information provided?	At diagnosis 52.4% (11)	During certain moments (transplantation/important immunosuppression) 19% (4)	At several moments during the treatment 23.8% (5)	At the request of families 4.8% (1)
Is the removal, even temporarily, of pets recommended?	Yes, all animals 18.2% (4)	Only some species 23.8% (5)	Only if the animal is sick 40.9% (9)	Never 18.2% (4)
How long is the pet removed after the cancer/immunodeficiency diagnosis?	For the entire duration of the treatment 26.7% (4)		Over a limited period 73.3% (18)	
How do you behave if a family requests to adopt a pet during the treatment?	We do not advise against 50% (11) but we suggest not adopting some species 23.8% (5)		Case-by-case assessment 13.6% (3)	We always advise against 36.4% (8)
Is the family advised to have a veterinary checkup for their pet?	Yes 100% (22)		No 0% (0)	
Are families advised not to let patients take care of the animals daily (e.g., changing the litter box, cleaning an aquarium, etc.)?	Yes 95.5% (21)		No 4.5% (1)	
Is the family advised to avoid animals outside the domestic environment (e.g., zoos, educational farms, etc.)?	For the entire duration of the treatment 36.4% (8)		Over a limited period 45.4% (10)	No 18.2% (4)
**Animal-Assisted Therapy**				
At your center, do you use AAI?	Yes 36.4% (8)		No 63.6% (14)	
Is it recommended to start AAI outside your center?	Yes 4.5% (1)		No 95.5% (21)	
Do household pets have access to your center at particular moments (e.g., terminally ill)?	Yes 31.8% (7)		No 68.2% (15)	
**Zoonoses**				
Have there been any cases of animal-related infections at your center?	Yes 18.2% (4)		No 81.8% (18)	
Was it possible to trace the source?	Yes, pet 25% (1)	Yes, animals outside the family 25% (1)	Yes, environmental origin 25% (1)	No 25% (1)
What consequences did such infections bring?	No complications 33.3% (1)	Hospitalization 66.7% (2)	Therapy delays 0% (0)	Death 0% (0)
Would you welcome a document for the management of zoonoses with indications to be given to patients in your center?	Yes 100% (22)		No 0% (0)	

## Data Availability

Not applicable.
